# Solvent-Induced Selectivity of Isoprene From Bio-Derived Prenol

**DOI:** 10.3389/fchem.2022.879129

**Published:** 2022-05-17

**Authors:** Jotheeswari Kothandaraman, Lelia Cosimbescu, Marie S. Swita

**Affiliations:** Pacific Northwest National Laboratory, Richland, WA, United States

**Keywords:** prenol, isoprene, selective catalysis, bio-derived, sustainability

## Abstract

In this work we demonstrate the selective catalytic conversion of prenol, which is an allylic alcohol that can be prepared from renewable resources to isoprene. The catalyst is an inexpensive molybdenum complex (Molyvan L) designed and used as an additive for lubricants. Isoprene is generated under relatively mild reaction parameters at 130–150°C, for 2 h, under vapor pressure conditions that do not exceed 50 psi. Two cases were studied: one in which Molyvan L was dissolved in a base oil at 1% concentration (weight/weight) and then mixed with a solvent and prenol and the other in which neat Molyvan L was introduced in the reaction and the base oil was replaced with the solvent and prenol. We investigated the selectivity of the reaction using the following solvents in both cases: dodecane, dodecanol, isododecane, octane, blendstock for oxygenate blending (BOB3), a fuel surrogate, a polyalphaolefin (PAO4), and methoxy polyethylene glycol (methoxy PEG350). Although conversion of prenol was above 94% in all experiments, isoprene was formed with various degrees of efficiency alongside a prenol isomeric alcohol, diprenyl ether and mixed ether via intramolecular and intermolecular dehydration reactions. Dodecane appeared to have the highest level of selectivity initially in base oil so we studied the effect of various dodecane-like solvents on isoprene yield and product profile. Surprisingly, octane (similar to dodecane) and isododecane (branched alkane) generated insignificant amounts of byproducts, essentially providing the highly desired isoprene with a very high selectivity. Branching of the solvent does not appear to have an effect on selectivity. Another advantage of this catalyst is the low loadings required to effect the transformation; that is, 0.25% (weight/volume) in the cases using neat Molyvan L and 0.5% (weight/volume) in the cases using Molyvan L dissolved in the base oil. Provided that prenol can be produced in large scale from bioresources, this work would enable the sustainable production of isoprene, in good yield, and with very high selectivity.

## 1 Introduction

Isoprene, an unsaturated hydrocarbon, is an important monomer used in the production of synthetic rubber (approximately 95% of isoprene produced is used for this purpose). It also is an important platform chemical used for a wide range of applications, such as fragrances, medicines, and pesticides. Currently, isoprene is mostly produced from fossil sources as a byproduct of the thermal cracking of oil or naphtha. About 800,000 metric tons are produced annually. With a strong industrial demand for isoprene, its sustainable production from raw materials derived from renewable sources is highly desired. (Ezinkwo et al., 2013).

Prenol (3-methyl-2-buten-1-ol) has been identified as an interesting and unusual oxygenated biofuel blendstock for use in spark ignition engines primarily because it exhibits a novel “octane hyperboosting” effect when blended with gasoline mixtures (Monroe et al., 2019). Because of its unique positioning of olefinic and alcohol functional groups, when blended with gasoline the research octane number (RON) of the mixture exceeds the performance of the individual pure components. This is in contrast with the well-known synergistic blending of oxygenates into gasoline, in which the measured RON never surpasses the RON of best performing component. In addition to reducing NO_x_ and soot production during combustion, which is typical of most alcohol-based fuels, prenol has low water miscibility, good energy density, high octane sensitivity, and low Reid vapor pressure. With the current trend of decarbonizing the transportation sector, including fuels and bio-derived oxygenates blending into fuels, there is even a greater incentive to produce prenol from biorenewable resources. Currently prenol is produced industrially as an intermediate for the generation of citral using petroleum-derived chemicals via a two-step process that first involves reacting isobutene and formaldehyde to form isoprenol and then isomerization of the isoprenol to prenol. (Bonrath and Netscher, 2005; Parker et al., 2016). Prenol is not naturally abundant, however, because of its promising fuel performance, researchers have studied its synthesis from renewable sources using metabolically engineered *Escherichia coli*. (Chou and Keasling, 2012; Zheng et al., 2013; George et al., 2015; Gupta and Phulara, 2015; Liang et al., 2021). With recent developments in biosynthetic approaches, prenol production of more than 2 g/L has been demonstrated, thus showing its potential as a next generation biofuel. (Zheng et al., 2013; Clomburg et al., 2019).

Previously, while studying the reactivity of prenol in the presence of Molyvan L (a molybdenum additive used as a friction modifier), we identified the major products of the reaction: 2-methylbut-3-en-2-ol, ethers, and isoprene (2-methyl-1,3-butadiene) (Cosimbescu et al., 2022). These moieties were produced via isomerization of prenol, intermolecular and intramolecular dehydrations, respectively, albeit with no selectivity to any specific product. Producing isoprene from bio-derived prenol by selective intramolecular dehydration is an attractive alternative route to the conventional petroleum-derived process. Bioproduction of isoprene has been gaining much attention recently, (Yang et al., 2012; Wang et al., 2017; Li et al., 2018; Li et al., 2019; Janke et al., 2020), and inspired by the body of work, we wanted to leverage our early results with prenol/Molyvan L catalyst and explore the potential of producing isoprene by selective intramolecular dehydration of 2-methylbut-3-en-2-ol, a tertiary isomeric alcohol of prenol that forms *in situ* in presence of Molyvan L.

In this work, we investigated conditions for the selective catalytic production of isoprene from prenol using Molyvan L as an inexpensive homogenous catalyst. Previous work published by us and by other research teams has shown that the selective production of isoprene from prenol is challenging under relatively mild conditions (<150°C) and often results in a mixture of products, such as ethers, ketones, and isomers of prenol in the presence of both homogenous and heterogeneous catalysts (Marton et al., 1989; Bianchini et al., 2001; Todd and Bielawski, 2013; Cosimbescu et al., 2022). Notably, Taylor and Shenk reported selective production of isoprene from prenol isomeric alcohol under harsh conditions (300°C) (Taylor and Shenk, 2002). However, in their study, the alcohol was prepared in 50% yield by the reduction of its butyne precursor, 2-methylbut-3-yne-2-ol, with a large excess of copper zinc dust. Several other products also were formed, presumably making isolation of the alcohol difficult. In contrast, our process is not only energy efficient (occurring at a lower temperature, low catalyst loadings, short reaction times), but can use “green” prenol produced from biomass. We have identified that the solvent plays a key role in controlling the selectivity of the product distribution <150°C. Furthermore, the optimized reaction conditions that provide high selectivity also would enable the separation of isoprene from the reaction mixture via distillation, as the minor products formed have much higher boiling points. To the best of our knowledge, this work is the first report of selective, catalytic, and high yielding production of isoprene directly from prenol under relatively mild reaction conditions and short reaction times.

## 2 Materials and Methods

### 2.1 Reagents

Prenol and isododecane (2,2,4,6,6-pentamethylheptane) were purchased from TCI America and used without further purification. Octane, methoxy PEG350 and dodecane were purchased from Aldrich and used as received. The blendstock for oxygenate blending (BOB3) was obtained from a commercial refiner and was used as received. BOB3 is a winter-blend, premium unadditized gasoline, with the lowest amount of butane among all available BOBs and therefore has the lowest vapor pressure. A detailed hydrocarbon analysis was conducted at Intertek Laboratory in accordance with ASTM D2699 to provide the BOB3 composition and carbon typing. The hydrocarbon group types in terms of total mass percent were as follows: paraffins—6.8225; isoparaffins—44.9437; olefins—8.5925; naphthenes—13.7524; aromatics—22.5572; c14+—0.7997; unknowns—2.5320. The fuel surrogate blend was prepared in house from the following components: isooctane (55%), heptane (15%), xylene (25%), 1-hexene (5%), and it is designed to mimic a gasoline fuel but with much fewer components. All components were reagent grade and purchased from Fisher.

Polyalphaolefin 4 (PAO4, provided by Exxon Mobil) was the base oil for Molyvan L additive/catalyst. PAO4 is a Group IV lubricant base stock with a kinematic viscosity around 4cSt at 100°C. Molyvan L [molybdenum di (2-ethylhexyl) phosphorodithioate] was obtained from Vanderbilt Chemicals LLC and was provided as a concentrated oil solution (75% w/w). We further diluted the received lubricant additive in PAO4 to obtain a 1% (w/w) solution. In the “neat” experiments, we used the molybdenum-based catalyst as received, in concentrated form.

### 2.2 Experimental Procedure

A 50 ml pressure vessel rated up to 1,000 psig from McMaster Carr was loaded with either: 5 ml prenol, 5 ml solvent (octane, dodecane isododecane, methoxy PEG 350, BOB3, fuel surrogate, or PAO4) and 10 ml of 1% Molyvan solution in PAO4; or 5 ml prenol, 15 ml of solvent as defined above, and 55–60 mg neat Molyvan L as the Mo catalyst. The reaction mixtures were premixed in a 20 ml scintillation vial to ensure homogeneity and take an aliquot that served as a baseline for the reaction. As such, the initial concentration of prenol was kept the same in both configurations, at 25% (v/v). The internal temperature was monitored via a handheld Omega thermometer attached to a type K thermocouple. A pressure gauge (McMaster Carr - part number: 9767T21) was attached to the side of the reaction vessel to monitor vapor pressures, typically between 40–50 psig. An external Omega type K thermocouple, attached to an over-temperature controller offset at 250°C, was kept inside the vessel heating mantle to prevent overheating. The mixtures were stirred at a temperature ∼150°C for 2 h, then chilled in ice as to prevent the loss of volatiles, particularly isoprene and placed in freezer.

### 2.3 Analysis

#### 2.3.1 Gas Chromatography *via* Flame Ionization Detection

Original (fresh, 0 h) and heated samples (2 h) were analyzed via GC-FID to quantify used prenol and generate conversions, as well as qualitatively compare the magnitude of isoprene peak from each reaction condition. Samples were analyzed using an Agilent 7,890 gas chromatograph equipped with a flame ionizing detector. The column was a DB5-MS 30 m × 250 μm x 1.0 µm film thickness with a carrier gas of helium flowing at 2.0 ml/min. The oven temperature was initially held for 1.0 min at 40°C and then ramped up at 10°C/min to a final temperature of 325°C, where it was held for 10 min. The injection volume of each neat sample was 1 µl and the inlet was heated to 260°C. Each sample was integrated using Agilent Data Analysis software. A baseline drop was used for integration to determine each peak area of compounds of interest. Using the peak areas, specific samples were compared to their respective originals at the beginning of the experiment. Chromatograms from each sample set (before and after heating) were overlaid to qualitatively inspect for area differences indicative of chemical changes with heating. All GC-FID overlays can be found in the SI.

### 2.4 Spectral Characterization

NMR spectra were obtained using a Bruker Avance NEO or Varian 500 MHz NMR Spectrometer console scanned at 500 MHz (^1^H). The chemical shifts are reported in delta (*δ*) units, parts per million (ppm) downfield from tetramethylsilane (TMS), Samples were prepared in deuterated chloroform containing dimethylterephthalate (DMTP) as an internal standard.

## 3 Results and Discussion

A simple process set-up comprised of a 50 ml pressure vessel rated up to 1,000 psig was loaded with either 5 ml prenol, 5 ml solvent (octane, dodecane, isododecane, methoxy PEG350, BOB3, fuel surrogate, or PAO4) and 10 ml of 1% Molyvan L solution in PAO4 (data shown in Supplementary Table S1) or 5 ml prenol, 15 ml of solvent as defined above, and 55–60 mg neat Molyvan L as the molybdenum catalyst (data shown in Supplementary Table S2).

**FIGURE 1:**
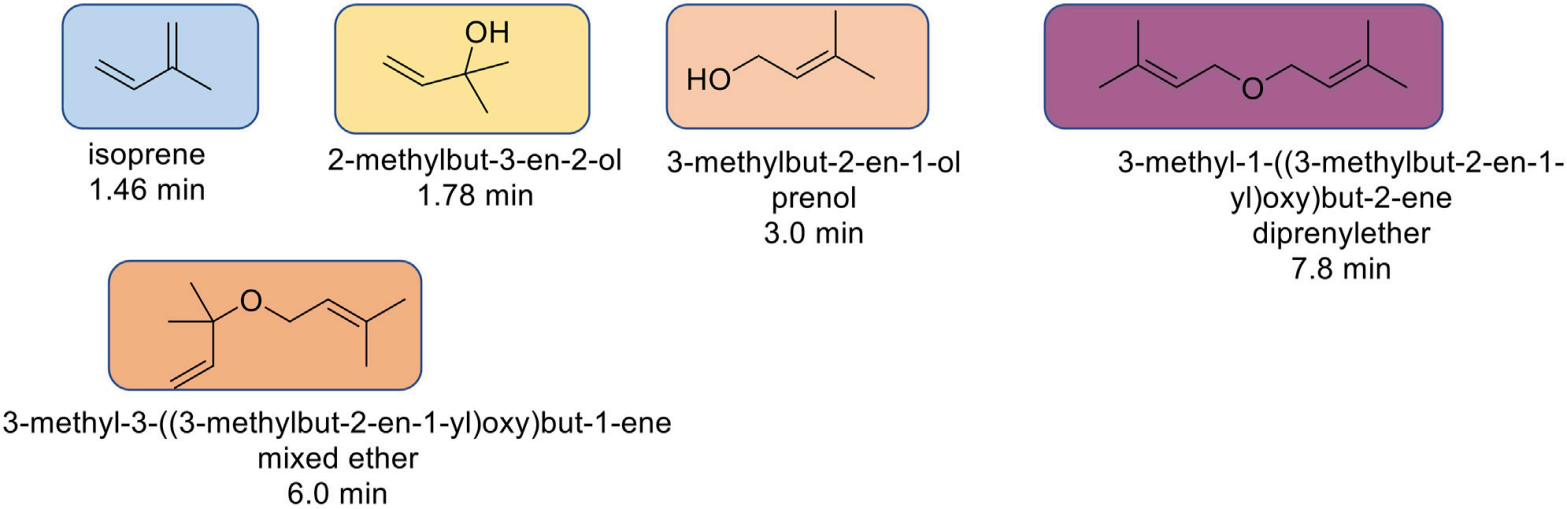
Structures of major products and their corresponding GC-FID retention times.

The reaction mixtures were premixed in a 20 ml scintillation vial to achieve homogeneity, and an aliquot of the mixture served as a baseline for the reaction. As such, the initial concentration of prenol was kept constant in both experiments, at 25% (volume/volume). Original (“fresh”, unheated, 0 h) and heated (2 h) samples were analyzed *via* gas chromatography-flame ionization detection (GC-FID) to quantify the percent of prenol used and generate conversions and to qualitatively compare the magnitude of isoprene peak from each reaction condition. The reaction profile was similar to others previously run, with four major products identified via GC-FID (Figure 1). Other minor unidentified species also were present, totaling no more than 10% of the total products.

**FIGURE 2:**
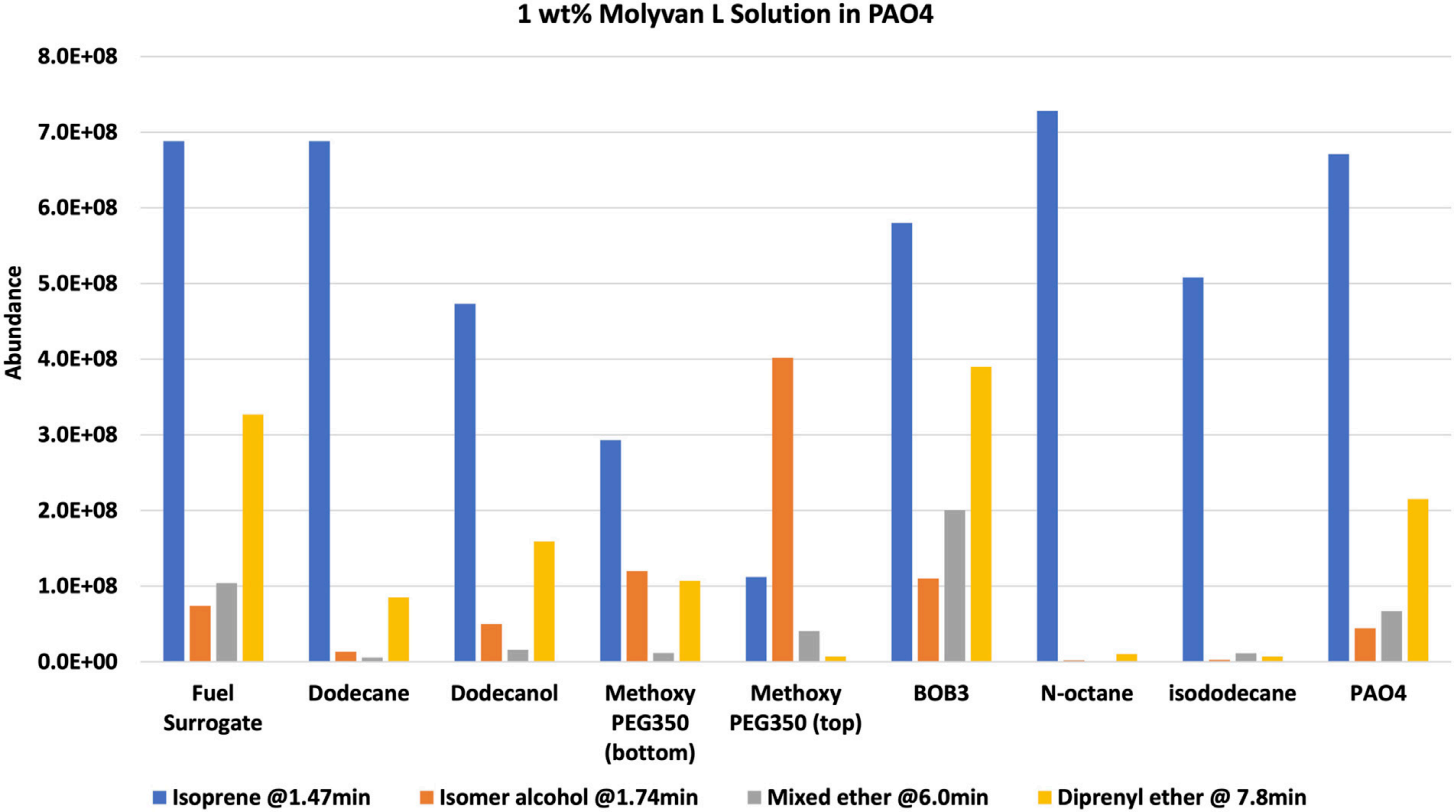
***1 wt% Molyvan L*** solution in PAO4. Reaction composition: 5 ml solvent, 5 ml prenol, 10 ml Molyvan L solution.

After the prescribed reaction time (2 h after reaching near 150°C), the reactor was chilled in ice to minimize the loss of the most volatile component, isoprene, and the mixture was transferred to a vial and then stored in a freezer until testing. The set-up has several metal hardware attachments (i.e., an inner temperature probe, a pressure gauge, and a rupture disk) of substantial volume where product(s) can condense and escape the final mixture, thus skewing the analytical results. The upper part of the set-up was not cooled, providing opportunities for product loss, particularly isoprene. The two configurations are referred to as 1% Molyvan L base oil solution and neat Molyvan L. These distinctively different conditions were implemented to probe for the highest conversion of prenol to products and for selectivity towards isoprene. Initial studies explored only lubricant containing systems and dodecane provided a promising result, that is why the study was continued in lubricant solvent-systems and expanded to systems without lubricant. The qualitative abundance data is shown in Figure 2 and Figure 4.

**FIGURE 3:**
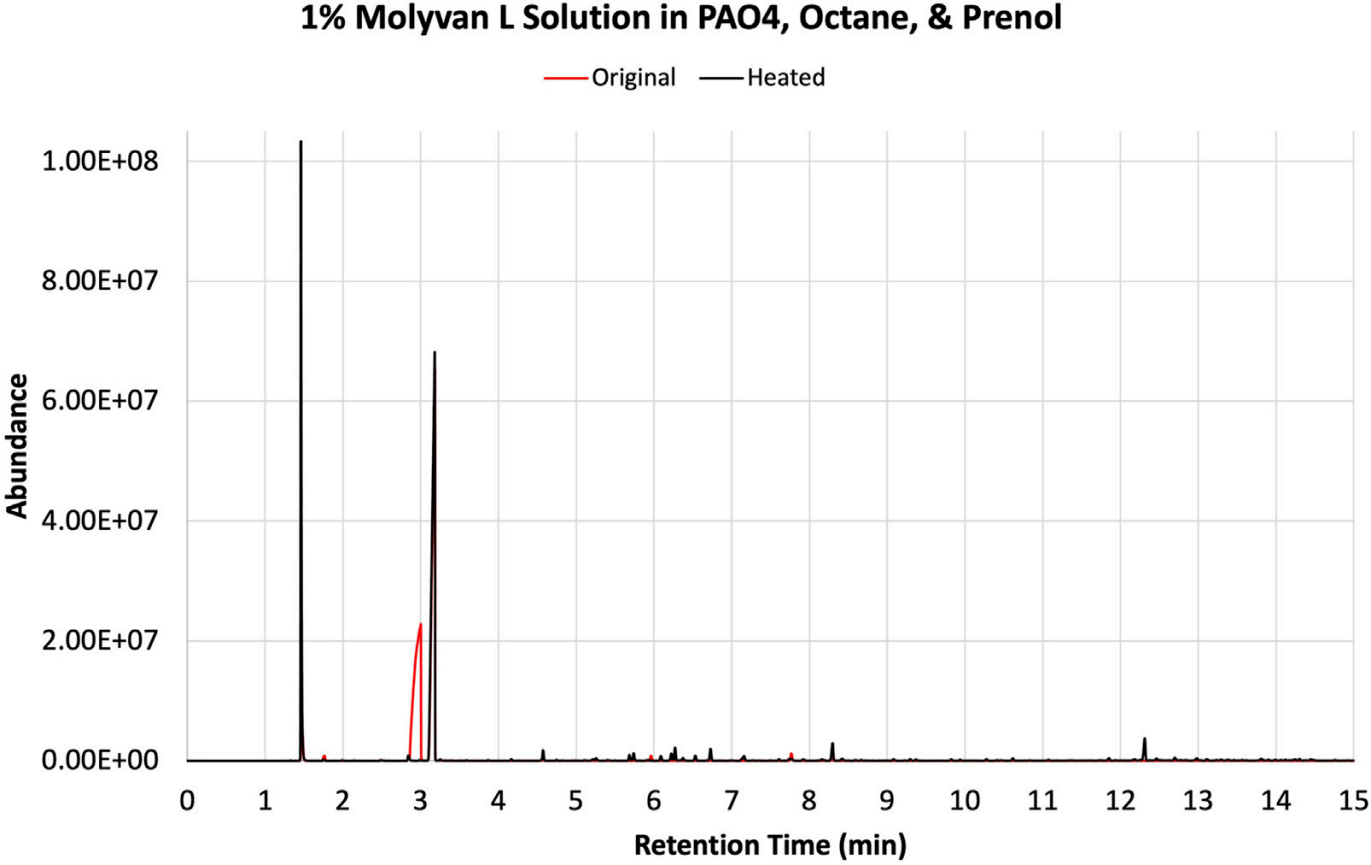
GC-FID traces of prenol, octane, and 1% Molyvan L base oil mixture before (red) and after (black) heating in a closed vessel.

The solvents screened are not typical organic solvents; rather, they are fuel-related solvents from which this research originated. As mentioned, it was discovered serendipitously that dodecane provided enhanced selectivity with a 1% Molyvan L base oil solution (Figure 2). While all solvent media tested provided near quantitative conversions of prenol, only few generated high amounts of isoprene. Dodecanol, which is slightly more polar than dodecane, was tested also, and although it yielded a substantial amount of isoprene, it also participated in dehydration reactions with itself and prenol and generated extra major byproducts aside from those shown in Figure 2. Therefore, the apparent enhanced selectivity for isoprene in this case is not real, as the graph ignores those byproducts only formed with dodecanol. Fuel surrogate and BOB3 generated large amounts of isoprene, as it qualitatively appears to be the largest product, but not selectively. We also investigated the methoxy PEG350 as a polar solvent, but we encountered challenges in assessing reaction efficiency towards isoprene formation because the solvent and products partition between the base oil and polar phases. A large amount of the isomeric alcohol was found in the bottom layer (aqueous, polar), so this reaction also did not provide the desired selectivity. Encouraged by the enhanced selectivity of dodecane, other high boiling-point solvents (required to minimize the vapor pressure of the system) such as octane and isododecane were explored. We were surprised to discover that both solvents provided very high selectivity towards the desired product with minimal formation of side products. The GC-FID of octane is shown as an example in Figure 3.

Although isododecane is expensive and is not a realistic solvent for a prospective industrial process, we chose it to study the effect of branching on selectivity. Both linear and branched systems have exhibited similar selectivity, showing the reaction profile is independent of solvent branching. In one example, PAO4 (a branched solvent system) was used as the solvent as well as the molybdenum catalyst medium to study its selectivity (Figure 2). Notably, PAO4 showed enhanced reactivity, as significant amounts (about 5% based on conversion of prenol compared to trace amounts for other solvent conditions) of isoprene and byproducts were formed in the “fresh” unheated sample even though it was placed in the freezer immediately after mixing.

**FIGURE 4:**
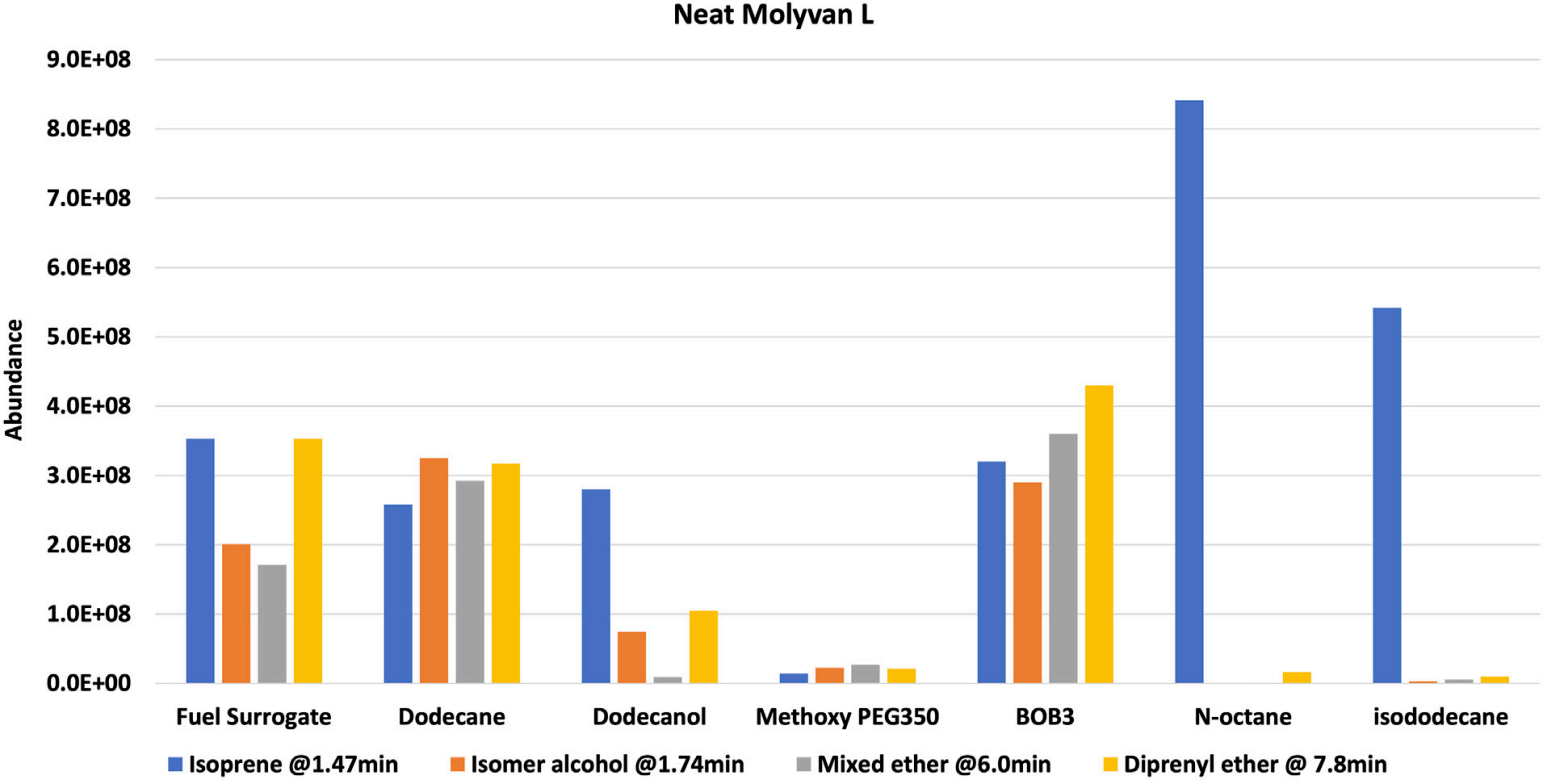
***Neat Molyvan L*** (75% in neutral oil). Reaction composition: 5 ml prenol, 15 ml solvent, Molyvan L (∼55–60 mg).

**FIGURE 5:**
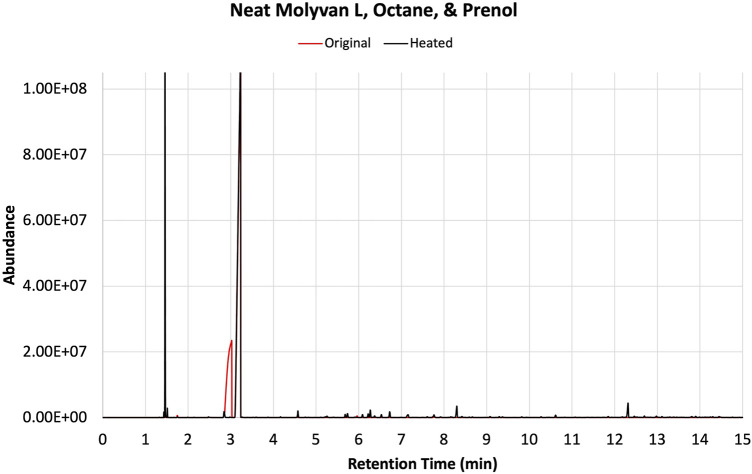
GC-FID traces of prenol, octane, and neat Molyvan L mixture before (red) and after (black) heating in a closed vessel.

Experiments were repeated with neat catalyst without a base oil, PAO4 (Figure 4). Overall, for this case, conversions and selectivity for isoprene appear slightly lower than those in the case in which a base oil was included (compare Supplementary Tables S1, S2). This is particularly true for the initially tested solvents (i.e., dodecane, fuel surrogate, BOB3, and methoxy PEG350). This is most evident in the case of dodecane, which yields much lower amounts of isoprene than the base oil containing counterpart (entries two in Supplementary Tables S1, S2). On closer examination of the data, the catalyst concentration in the overall *neat* mixture was about half of that used in the lubricant mixture. The lower conversions could be attributed to this discrepancy; however, the product profile and isoprene selectivity are unlikely to be affected by the lower catalyst amounts. In contrast, octane and isododecane (Figure 4) show remarkable selectivity even when neat Molyvan L was used. The octane product profile is shown as an example in Figure 5.

Notably, these solvents promoted enhanced reactivities even at cold temperatures (freezing and room temperatures), as a significant amount of isoprene was formed in the “fresh” samples. It is important to note that although BOB3 was included as a (co)solvent in the testing matrix, even if it provided a stellar selectivity for isoprene, it would not be a useful or practical solvent system, as isoprene could not be isolated due to the complex make-up of this fuel blend and the presence of many light fractions that would co-distill with isoprene. In contrast, all other mixtures could be easily subjected to distillation to isolate the lowest boiling point fraction, isoprene. In all examples from Figures 2, 4 a GC-FID peak area discrepancy from the prenol consumed and the major products formed suggests mass losses. About 10% of the mixtures are made up of unidentified species that are present in much smaller amounts than the byproducts of interest. All things considered, there still appears to be ∼20–25% area loss from prenol to products, albeit GC-FID is not a quantitative technique. Isoprene is the lightest fraction of the reaction mixture at 1.47 min and therefore is the most likely component to escape the mixture during transfers, transport, and analysis. Although 2-methylbut-3-en-2-ol (the isomer of prenol) appears close in retention time to isoprene at 1.76 min, its boiling point is substantially higher than isoprene, at 99 versus 34°C. To confirm our suspicions, an experiment mimicking the 1 wt% Molyvan L solution in PAO4 conditions was conducted, in which 10 ml of PAO4, 5 ml of octane, and 2.7 ml of isoprene were heated at ∼145–150°C for 2 h. Aliquots before and after heating were analyzed by ^1^HNMR to quantify losses during heating (Supplementary Figures S18, S19). Isoprene quantification before and after heating against an internal standard (dimethylterephthalate) revealed a 12% loss as a result of heating alone, not accounting for equal losses during transfers and weighing (2.03 mmol isoprene per gram of solution before heating versus 1.78 mmol of isoprene per gram of solution after heating).

To quantitatively evaluate the amount of isoprene in the final mixtures, albeit after storage in the freezer for several weeks, we conducted ^1^HNMR analysis using the procedure described below. First, an aliquot (∼0.2 g) from the reaction mixture was combined with a CDCl_3_ solution (∼0.6 g) containing a known amount of internal standard (dimethylterephthalate). This internal standard has well resolved singlet peaks (8.1 and 3.9 ppm) against the isoprene signature peaks (6.48–6.42 ppm) and away from other olefinic byproducts. A weight measurement is more accurate than a volume measurement and it enables integration of proton peaks of these constituents and therefore quantification of the amount of isoprene present in the mixture. The two selected experiments for this study were those with octane solvent that gave the largest selectivity. Details of this procedure are found in the SI. The yields of isoprene from entries six in Supplementary Tables S1, S2 derived both from ^1^HNMR and GC-FID analyses are shown in Table 1.

**TABLE 1:** Yields of isoprene from entry six of Supplementary Tables S1, S2 derived both from ^1^HNMR and GC-FID analyses.

Experiment	Yield From ^1^HNMR (%)	Apparent Yield From GC-FID (%)
Octane (1 wt% Molyvan L in base oil, entry 6 in Supplementary Table S1)	54	44
Octane (neat Molyvan L, entry 6 in Supplementary Table S2)	69	52

**FIGURE 6:**
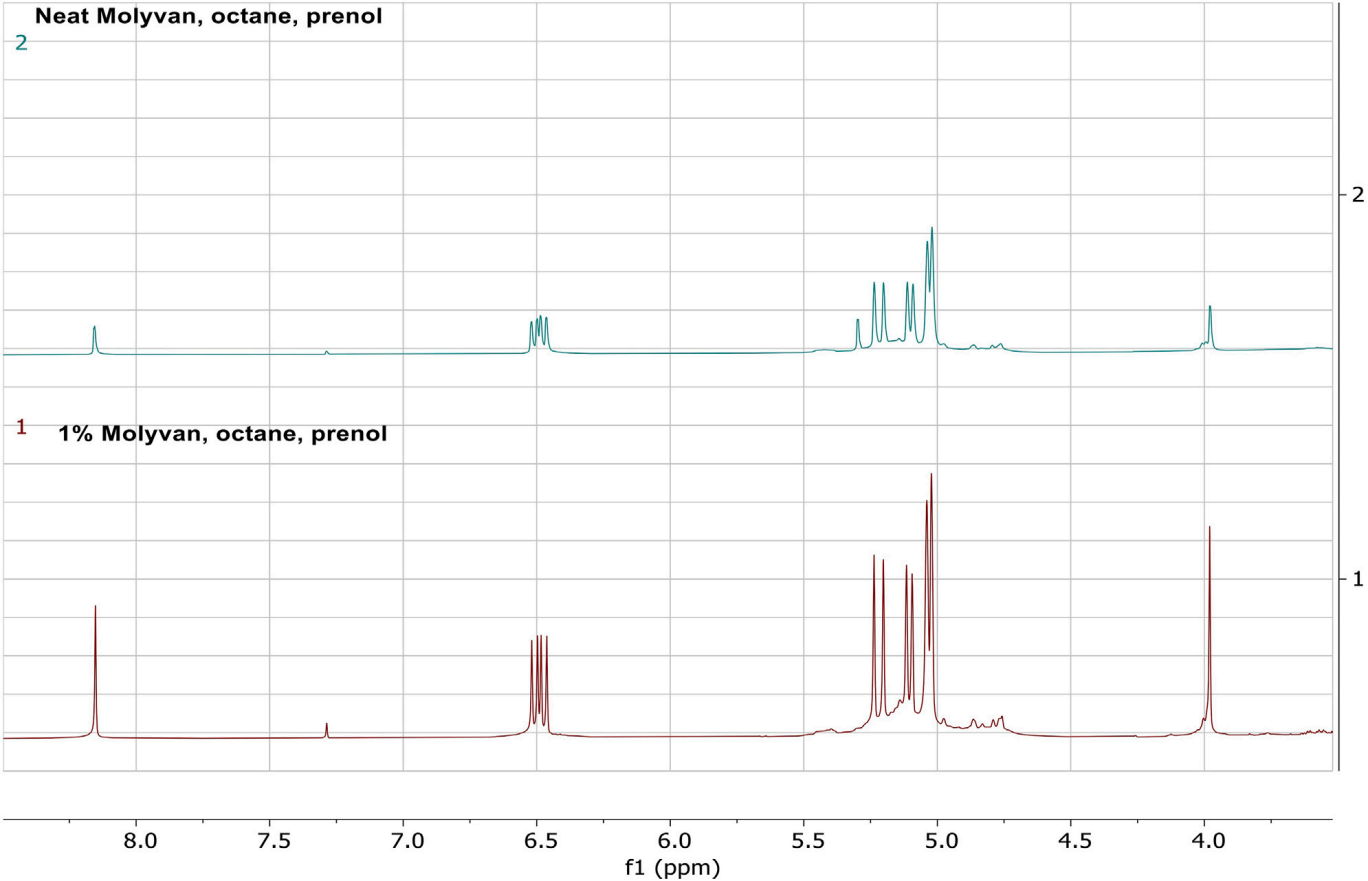
Quantification of isoprene present in the octane reactions via ^1^HNMR.

The stacked ^1^HNMR results of the two reaction mixtures demonstrate the similarity of the mixtures, the absence of isomeric alcohol that appears at 5.9 ppm, but presence of other olefinic species that partly overlap with the isoprene peaks at ∼5 ppm (Figure 6).

**FIGURE 7:**
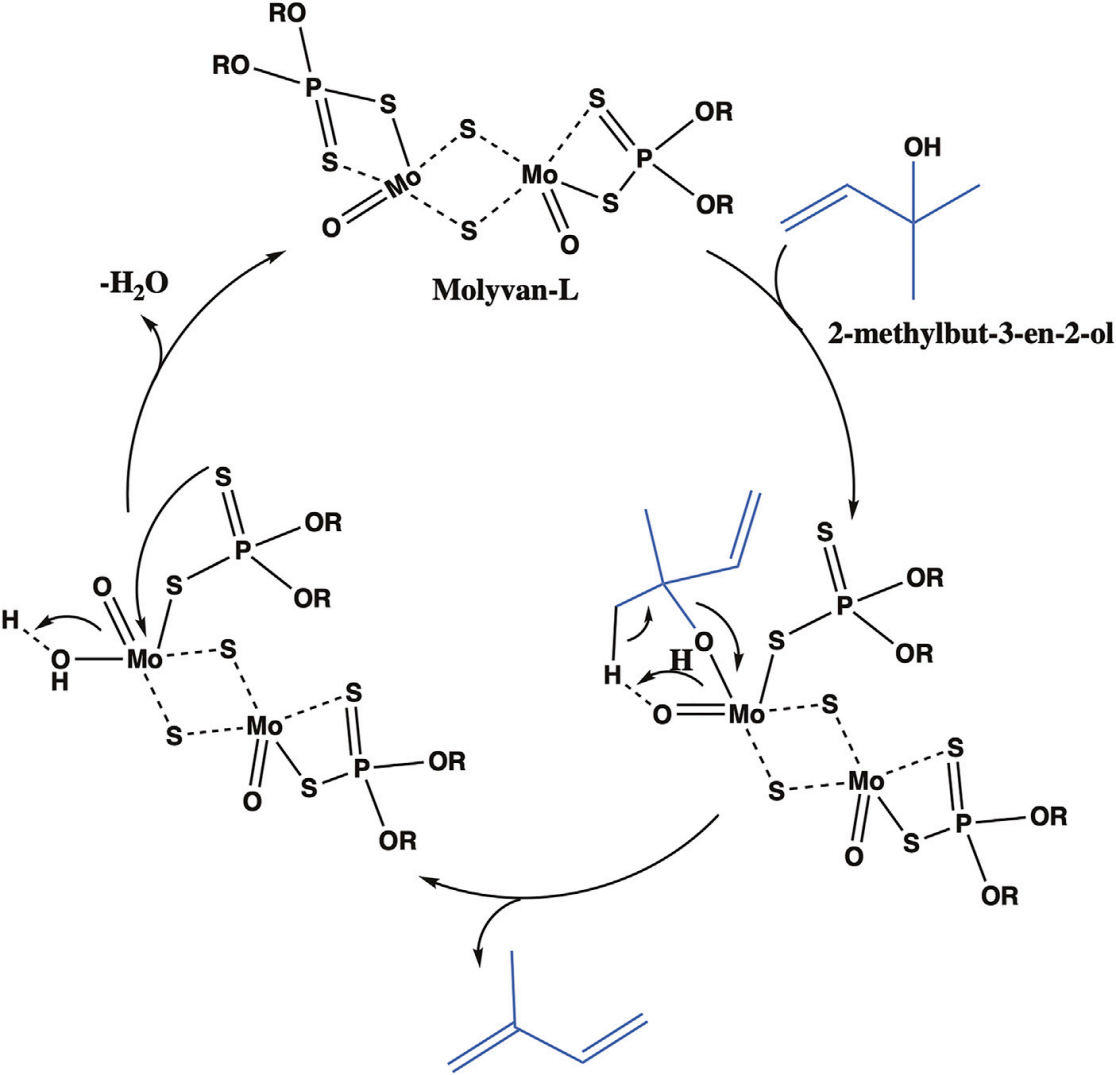
Proposed catalytic cycle for the isoprene formation from the *in situ* formed isomeric alcohol.

Previous attempts by others to synthesize isoprene or ethers from prenol resulted in low yields and poor selectivity. For example, Bielawski et al. studied a graphite oxide activated zeolite catalyst at 150°C for selective dehydration of different alcohols, including prenol, to corresponding alkenes (Todd and Bielawski, 2013). Although some alcohols formed olefins with good conversion and selectivity, prenol or the isomeric alcohol formed isoprene with poor yields and selectivity. Similarly, while attempting to form carbonyl compounds by isomerization of allylic alcohols at 100°C, Bianchini et al. reported that prenol formed a range of products including isoprene but with poor yields and selectivity (Bianchini et al., 2001). The reaction mechanism and the conditions controlling the selectivity of dehydration of prenol or the isomeric alcohol is of fundamental interest. As mentioned earlier, we serendipitously identified that by simply choosing the right solvent, isoprene can be selectively formed from prenol (isomerization followed by elimination), thus suppressing ether formation (substitution). Strong protic or Lewis acid catalysts have been reported to be active for allylic rearrangements and dehydration reactions (Overman et al., 1978; Moser, 1989; Larsen et al., 1997; Jacob et al., 1998; Matthias Kiefer et al., 1998; Korstanje et al., 2012). Molyvan-L, a strong Lewis acid catalyst, is likely to catalyze an allylic rearrangement of prenol to a more stable and substituted tertiary alcohol (i.e., the isomeric alcohol), (Belgacem et al., 1992; Jacob et al., 1998; Matthias Kiefer et al., 1998; Fronczek et al., 2002), which then can undergo intramolecular dehydration to form isoprene (Figure 7). A reaction mechanism for isoprene formation from *in situ* formed isomeric alcohol has been proposed based on previously reported oxo complexes (Korstanje et al., 2012; Korstanje et al., 2013). The Lewis acidic Molyvan-L coordinates with the isomeric alcohol to form molybdenum-alkoxy species, which then expels isoprene to form a hydroxy molybdenum species. Upon expulsion of water, the original Molyvan-L catalyst is regenerated for subsequent catalysis.

As shown in many entries in Supplementary Tables S1, S2, the strong Lewis acidic Molyvan-L also can coordinate with multiple alcohols and catalyze the intermolecular dehydration of prenol to ethers but to a significantly lesser extent in octane and isododecane solvents. Notably, that significant reactivity difference is observed even between two simple straight chain alkanes, dodecane (entry two in Supplementary Table S2) and octane (entry six in Supplementary Table S2). The difference in the boiling point and vapor pressure between these solvents could influence the product distribution. At a reaction temperature of 150°C, in the case of octane (boiling point 125.6°C), the prenol isomer alcohol concentration in the condensed phase is expected to be higher as opposed to dodecane (boiling point 216.2°C), which could influence the subsequent product distribution. At this point, it is not very clear how the solvent affects the selectivity to one product over the other. *In situ* reactivity studies could shed light on the reaction mechanism. In addition to the aforementioned direct formation of isoprene from prenol via isomer alcohol dehydration, isoprene formation from elimination of ethers could not be excluded (Al-Faze et al., 2021).

Overall, the process is expected to be easily scalable, provided the reaction is run in a vessel capable of sustaining the vapor pressure of the reaction medium. A qualitative analysis of the most selective process, octane with or without PAO4, appears amenable for scale-up. The reagents are inexpensive or recyclable. Molyvan L was used at 0.25% (weight/volume) concentration in the neat reaction condition, and reaction optimization may lower that amount further. PAO4 is a commodity chemical and costs about $1/pound. N-octane is not inexpensive; however, more work may reveal that 99% reagent grade is not necessary to achieve high reactivity, and a lower grade solvent may be sufficient. The octane should be recoverable in the isoprene isolation process, via fractional distillation, or after isoprene removal, or the remaining mixture could be recycled back through another reaction cycle. There are many adjustments that could be made to optimize the process, reduce energy requirements, and improve cost. We have only laid the foundation for future opportunities.

## 4 Conclusion

We have reported herein a simple, efficient, and selective synthesis of isoprene from prenol in the presence of a commercially available molybdenum catalyst. We serendipitously found that the choice of solvent plays a key role in suppressing the formation of side products such as ethers. Among the solvents tested, the use of simple long chain alkanes such as octane but also highly branched alkanes such as isododecane resulted in high conversion and selectivity to isoprene. In the case of octane, the highest yield was obtained with neat Molyvan L, in the absence of PAO4, at 69% yield (and quantitative conversion of prenol). Selectivity is very high at ∼90% based on results from our GC-FID analysis. Discerning the reaction mechanism could provide additional insights and understanding of the catalytic cycle and help to optimize the production of isoprene under mild conditions.

## Data Availability

The original contributions presented in the study are included in the article/Supplementary Material, further inquiries can be directed to the corresponding author.
