# Supplementary material for: Solvent-Induced Selectivity of Isoprene From Bio-Derived Prenol
Source: Front Chem. 2022 May 17;10:879129. doi: 10.3389/fchem.2022.879129 (PMC9152107; doi:10.3389/fchem.2022.879129)
Supplement: Supplementary file 1 [file Table1.DOCX]

Supplementary Material

**Table S1**. ***1 wt% Molyvan L solution in PAO4***. Reaction composition: 5 mL solvent, 5 mL prenol, 10 mL Molyvan L solution.

| Entry # | solvent | Original  prenol | After heating prenol | Isoprene @1.47min | Isomer alcohol @1.74min | Mixed ether @6.0min | Diprenyl ether  @7.8min | Prenol conversion |
| --- | --- | --- | --- | --- | --- | --- | --- | --- |
| **1** | Fuel Surrogate | 1.43x10^9^ | 2.20x10^7^ | 6.88x10^8^ | 7.4x10^7^ | 1.04x10^8^ | 3.27x10^8^ | 98% |
| **2** | Dodecane | 1.38x10^9^ | 0.417x10^7^ | 6.88x10^8^ | 1.31x10^7^ | 0.546x10^7^ | 8.5x10^7^ | 99% |
| **3** | Dodecanol | 1.54x10^9^ | 1.50x10^7^ | 4.73x10^8^ | 5.0x10^7^ | 1.56x10^7^ | 1.59x10^8^ | 99% |
| **4** | Methoxy PEG350  Bottom layer |  | 5.57x10^7^ | 2.93x10^8^ | 1.2x10^8^ | 1.17x10^7^ | 1.07x10^8^ | n/a |
| **4’** | Methoxy PEG350  Top layer |  | 1.09x10^7^ | 1.12x10^8^ | 4.02x10^8^ | 4.04x10^7^ | 0.68x10^7^ | n/a |
| **5** | BOB3 | 1.55x10^9^ | 1.6x10^7^ | 5.8x10^8^ | 1.1x10^8^ | 2.0x10^8^ | 3.9x10^8^ | 99% |
| **6** | N-octane | 1.63x10^9^ | 1.13x10^7^ | 7.28x10^8^ | 0.193x10^7^ | 0.017x10^7^ | 1.02x10^7^ | 99% |
| **7** | isododecane^*^ | 1.39x10^9^ | 0.25x10^7^ | 5.08x10^8^ | 0.25x10^7^ | 1.14x10^7^ | 0.68x10^7^ | 100% |
| **8** | PAO4 | 1.85x10^9^ | 1.85x10^7^ | 6.71x10^8^ | 4.45x10^7^ | 6.67x10^7^ | 2.15x10^8^ | 99% |

^*^proper name: 2,2,4,6,6-pentamethylheptane

**Table S2**. ***Neat Molyvan L*** (75% in neutral oil). Reaction composition: 5 mL prenol, 15 mL solvent, Molyvan L (~55–60 mg).

| Entry# | Solvent | Original  prenol | After heating  prenol | Isoprene @1.47min | Isomer alcohol @1.74min | Mixed ether @6.0min | Prenyl ether @7.8min | Prenol Converted |
| --- | --- | --- | --- | --- | --- | --- | --- | --- |
| **1** | Fuel Surrogate | 1.47x10^9^ | 3.88x10^7^ | 3.53x10^8^ | 2.01x10^8^ | 1.71x10^8^ | 3.53x10^8^ | 97% |
| **2** | Dodecane | 1.46x10^9^ | 8.48x10^7^ | 2.58x10^8^ | 3.25x10^8^ | 2.92x10^8^ | 3.17x10^8^ | 94% |
| **3** | Dodecanol | 1.2x10^9^ | 2.65x10^7^ | 2.8x10^8^ | 7.46x10^7^ | 0.89x10^7^ | 1.05x10^8^ | 98% |
| **4** | Methoxy PEG350 | 8.23x10^8^ | 2.3x10^8^ | 1.42x10^7^ | 2.24x10^7^ | 2.68x10^7^ | 2.14x10^7^ | 97% |
| **5** | BOB3 | 1.62x10^9^ | n/a | 3.2x10^8^ | 2.9x10^8^ | 3.6x10^8^ | 4.3x10^8^ | Not evaluated due to peak overlap |
| **6** | n-Octane | 1.61x10^9^ | 0.19x10^7^ | 8.41x10^8^ | 0.019x10^7^ | 0.086x10^7^ | 1.62x10^7^ | 100% |
| **7** | isododecane | 1.60x10^9^ | 0.45x10^7^ | 5.42x10^8^ | 0.27x10^7^ | 0.54x10^7^ | 0.98x10^7^ | 100% |

**GC-FID overlays**

The black plots are post reaction, labeled as 2h, while the red plots are original, labeled as 0h. In most cases a zoomed version it is shown up to 10-15min, to better see region where peaks of interest are revealed.


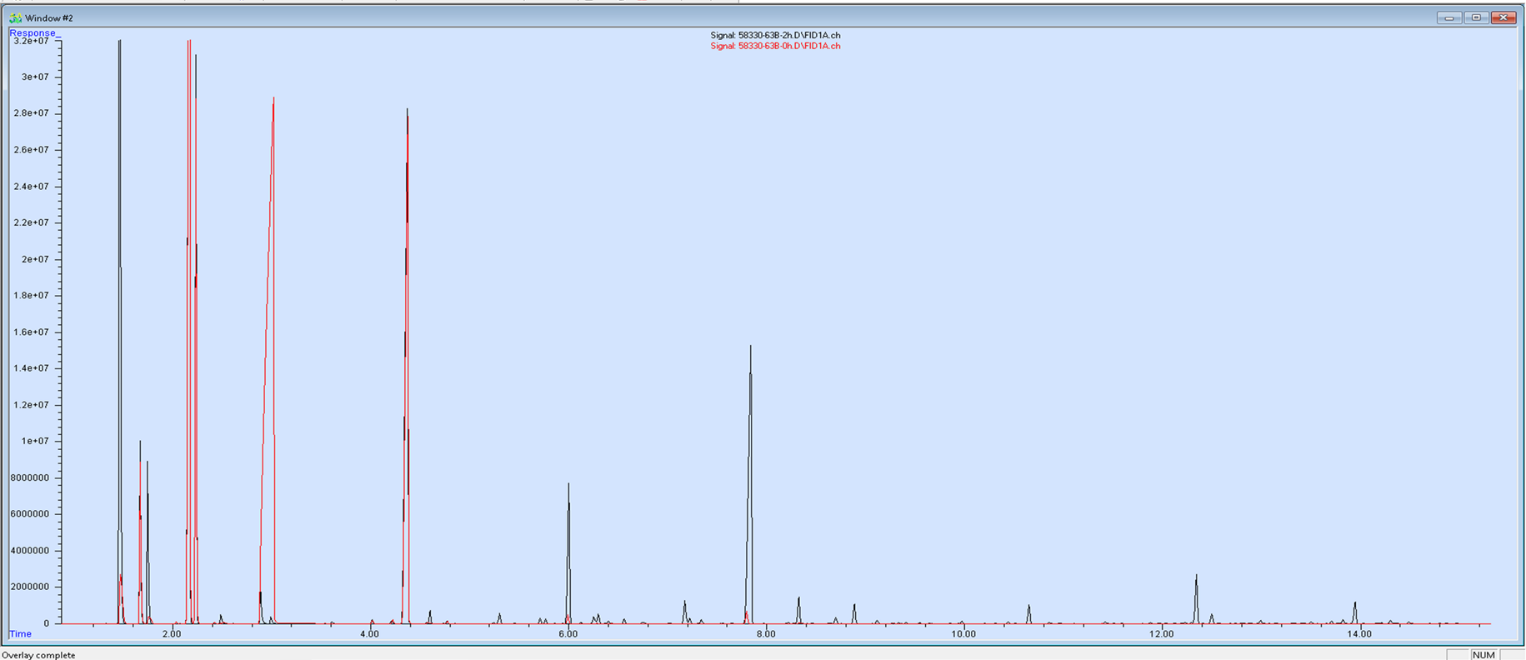


**Figure S1**: Reaction mixture from entry 1, Table 1 (1% Molyvan and fuel surrogate)


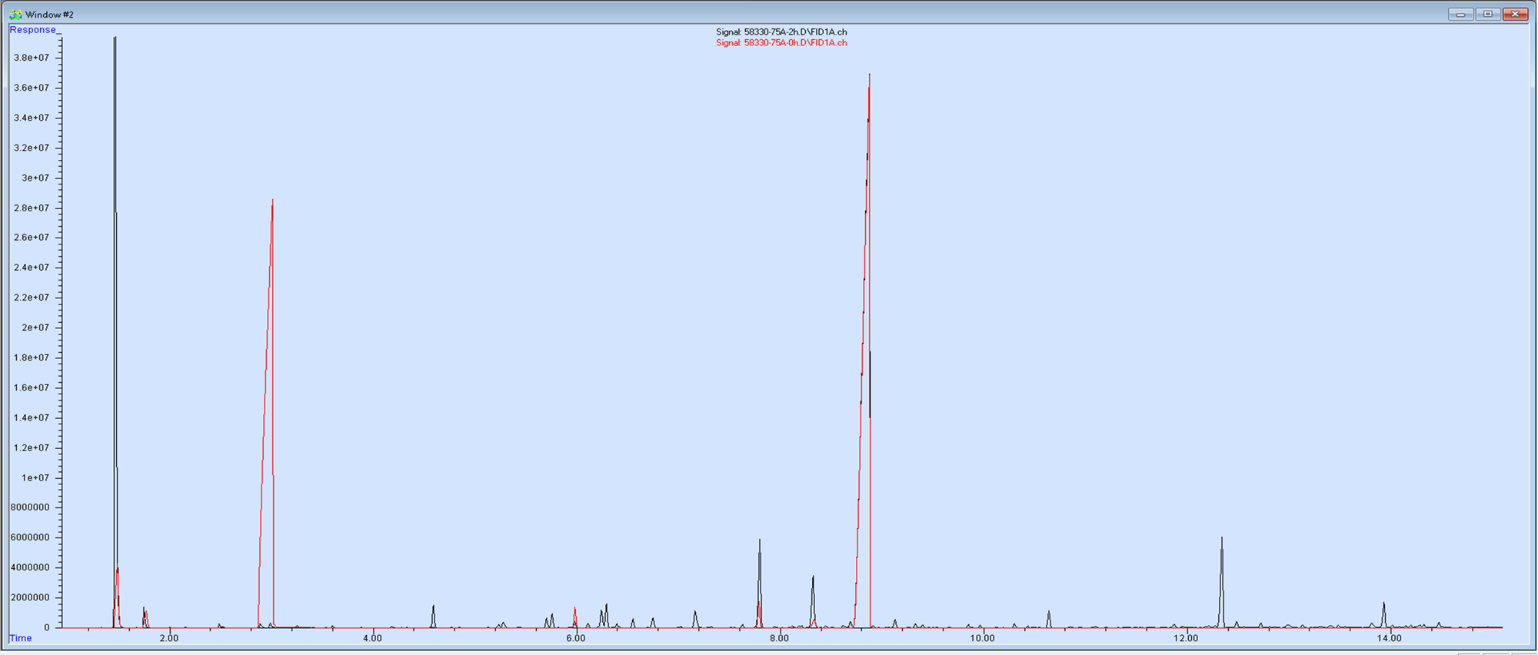


**Figure S2**: Reaction mixture from entry 2, Table 1 (1% Molyvan L and dodecane)


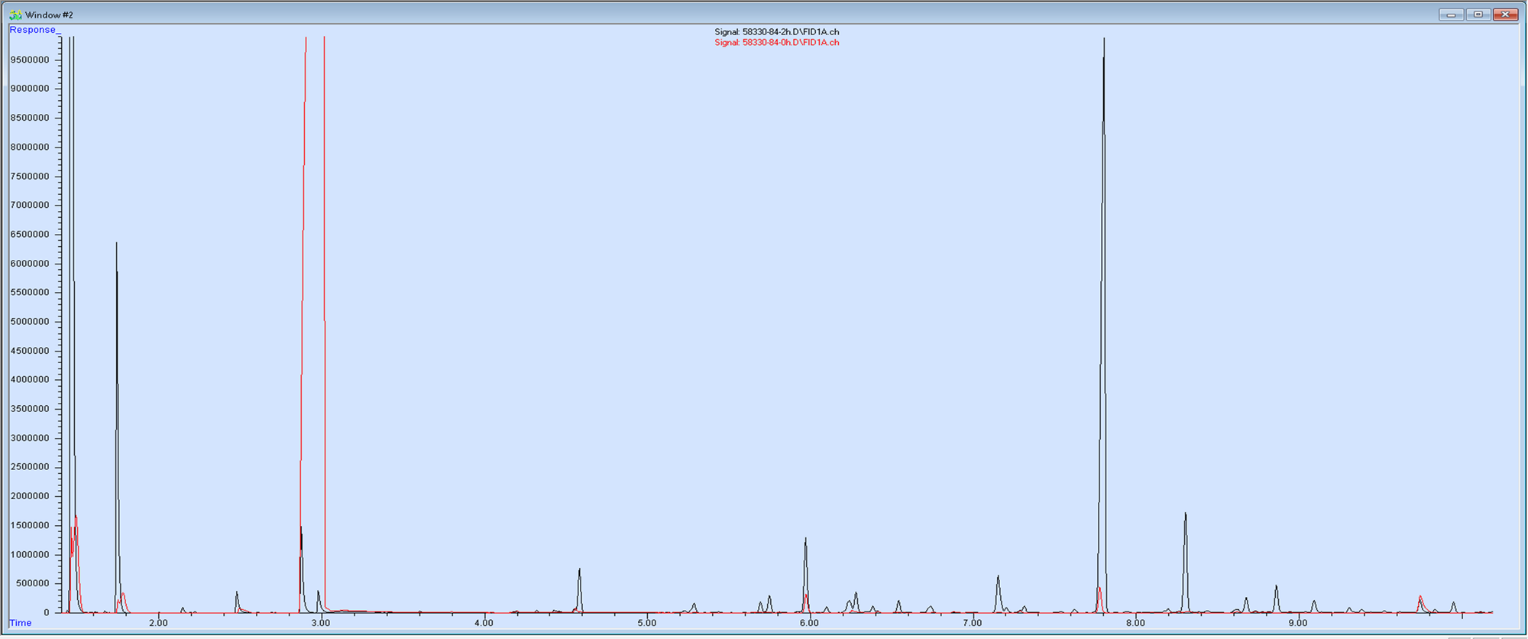


**Figure S3**: Reaction mixture from entry 3, Table 1 (1% Molyvan L and dodecanol)


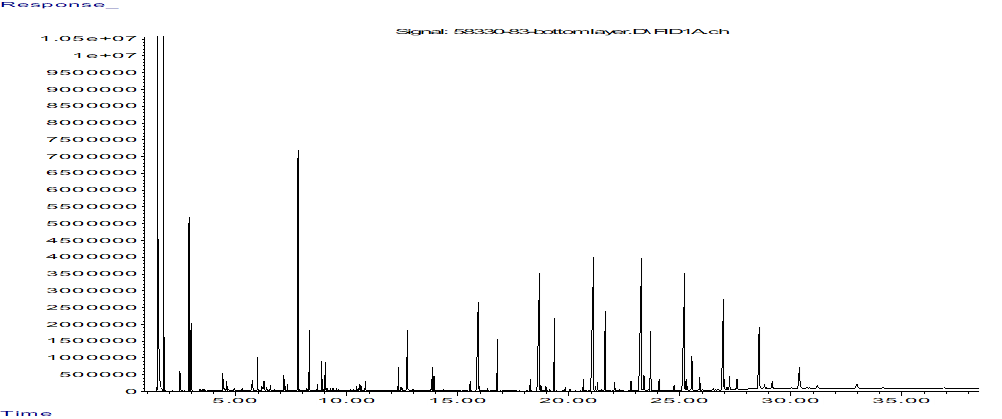


**Figure S4**: Reaction mixture from entry 4, Table 1 (1%Molyvan L and methoxy PEG350 bottom layer).


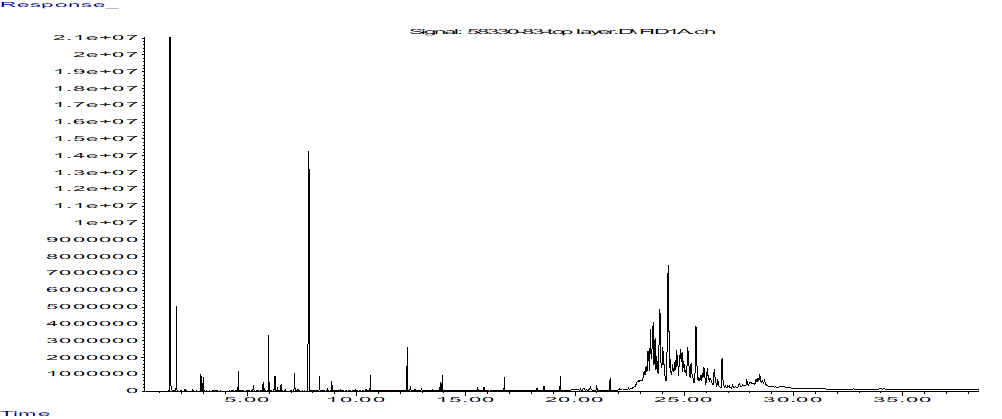


**Figure S4’**: Reaction mixture from entry 4, Table 1 (1%Molyvan L and methoxy PEG350 top layer).


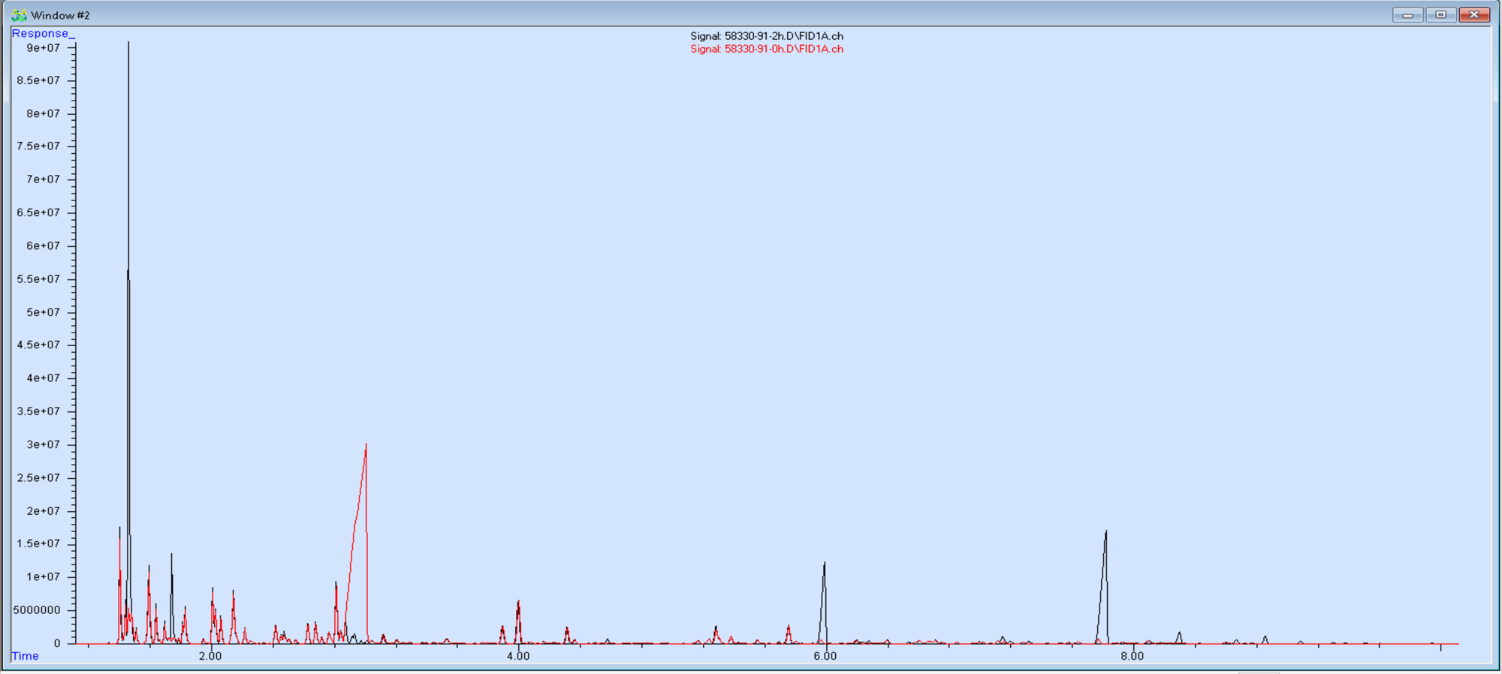


**Figure S5**: Reaction mixture from entry 5, Table 1 (1% Molyvan L and BOB3)


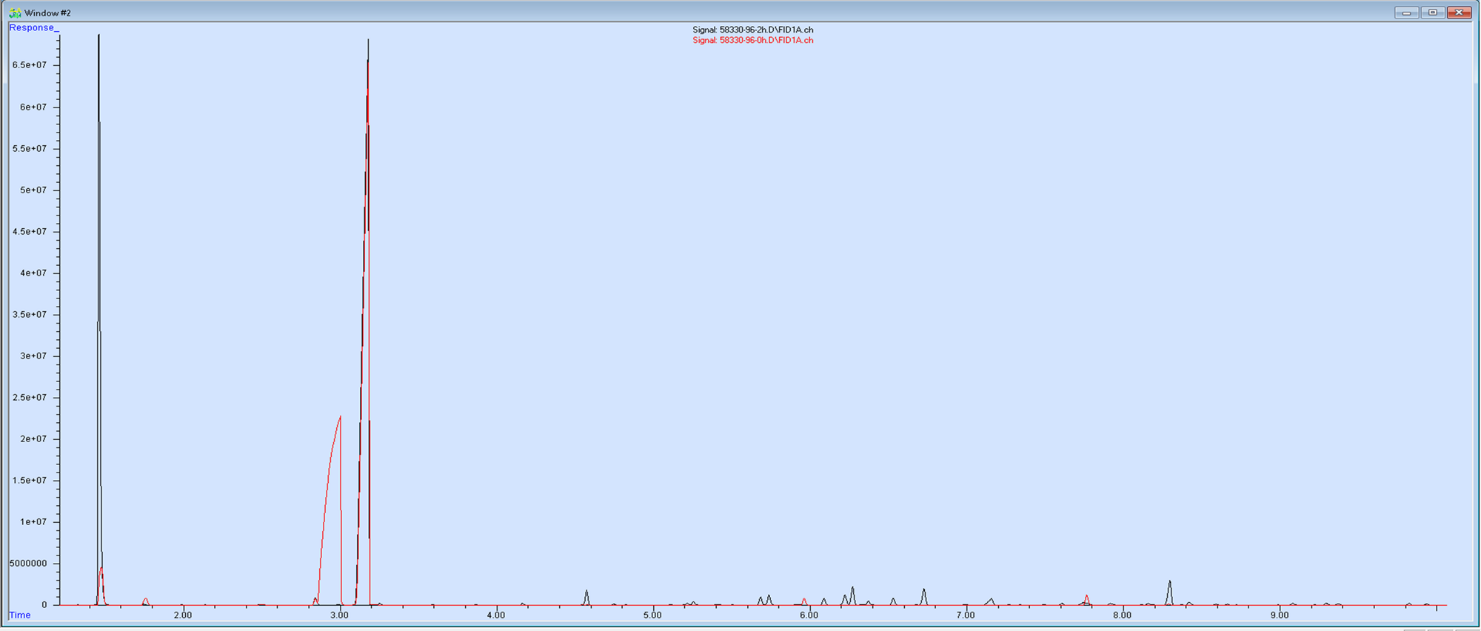


**Figure S6**: Reaction mixture from entry 5, Table 1 (1% Molyvan L and octane)


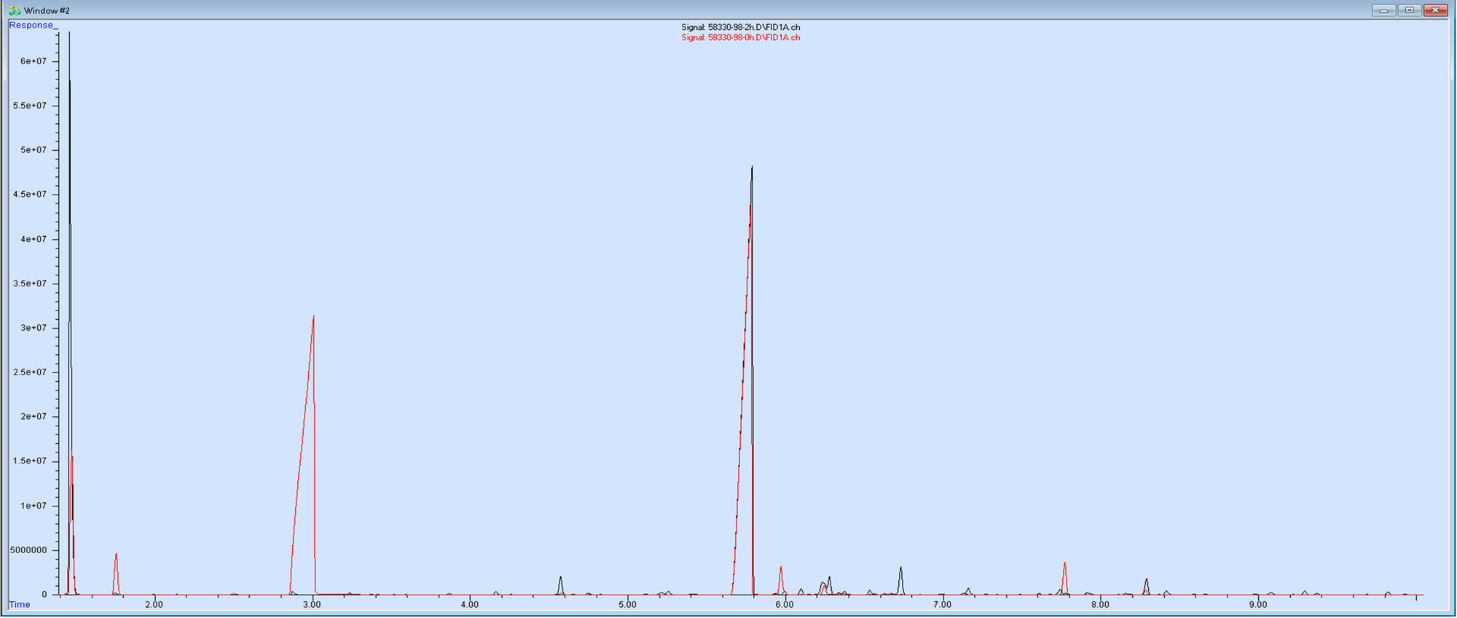


**Figure S7**: Reaction mixture from entry 7, Table 1 (1% Molyvan L and isododecane)


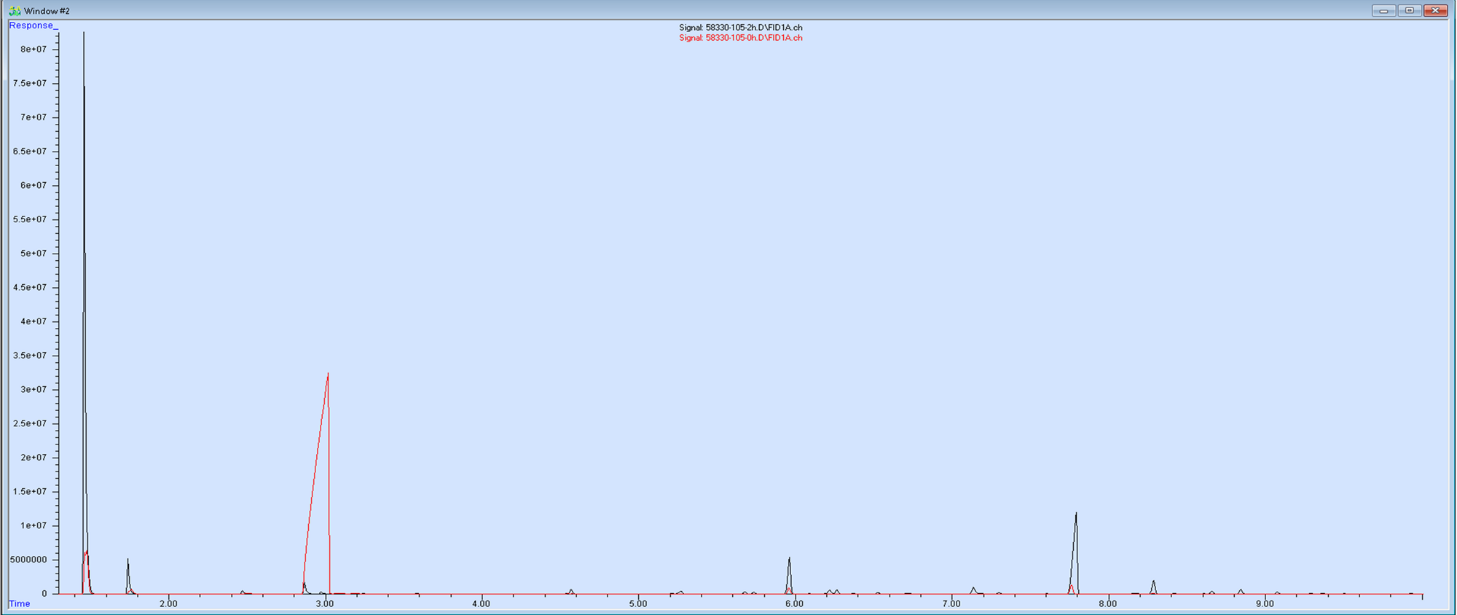


**Figure S8**: Reaction mixture from entry 8, Table 1 (1% Molyvan L and PAO4)


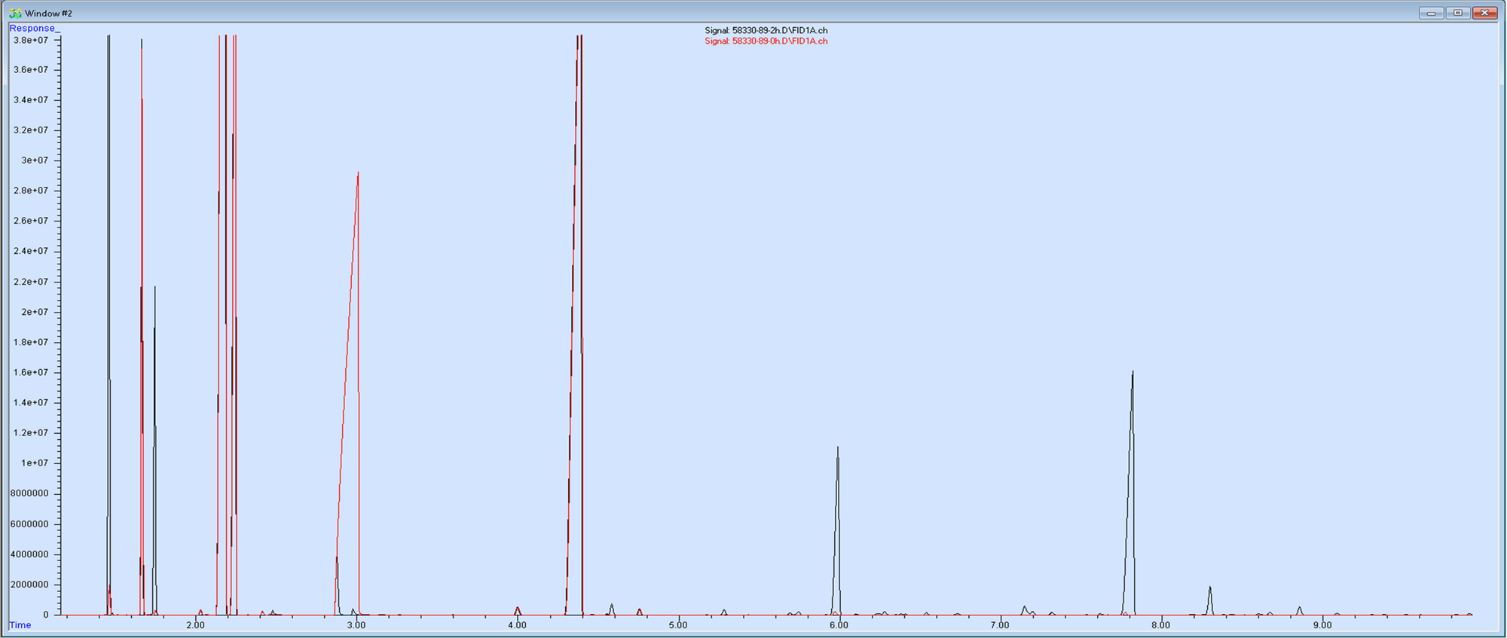


**Figure S9**: Reaction mixture from entry 1, Table 2 (neat Molyvan L and fuel surrogate)


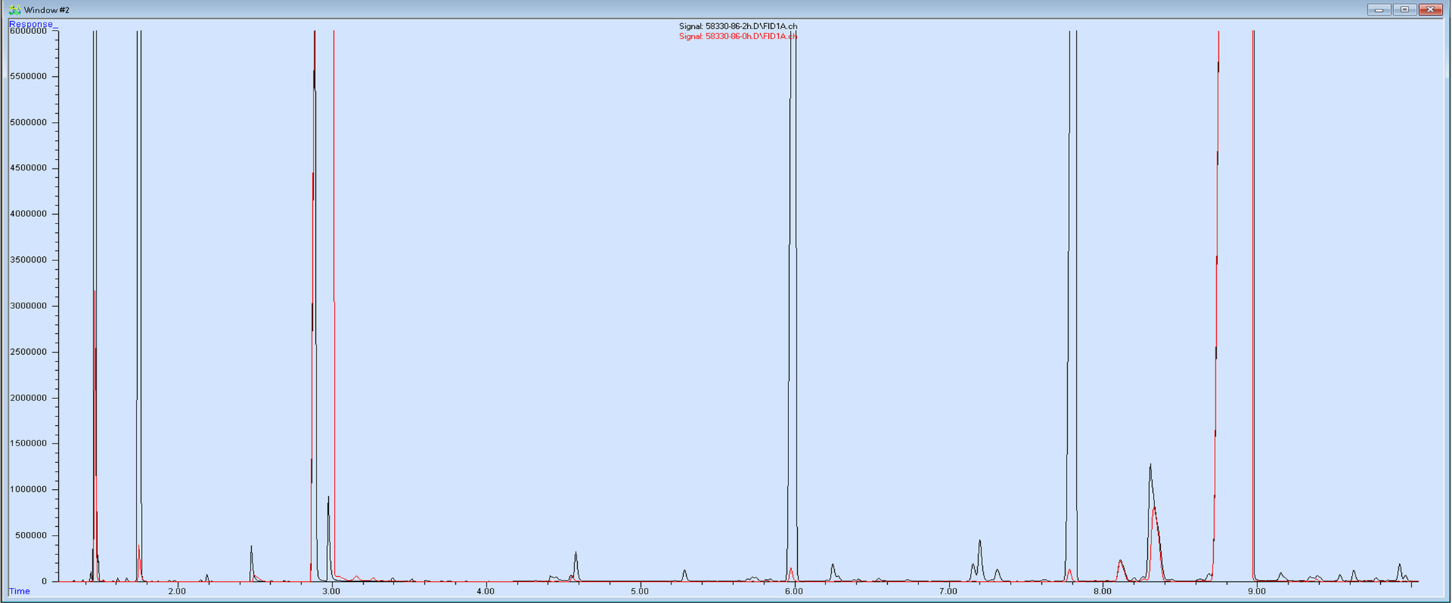


**Figure S10**: Reaction mixture from entry 2, Table 2 (neat Molyvan and dodecane)


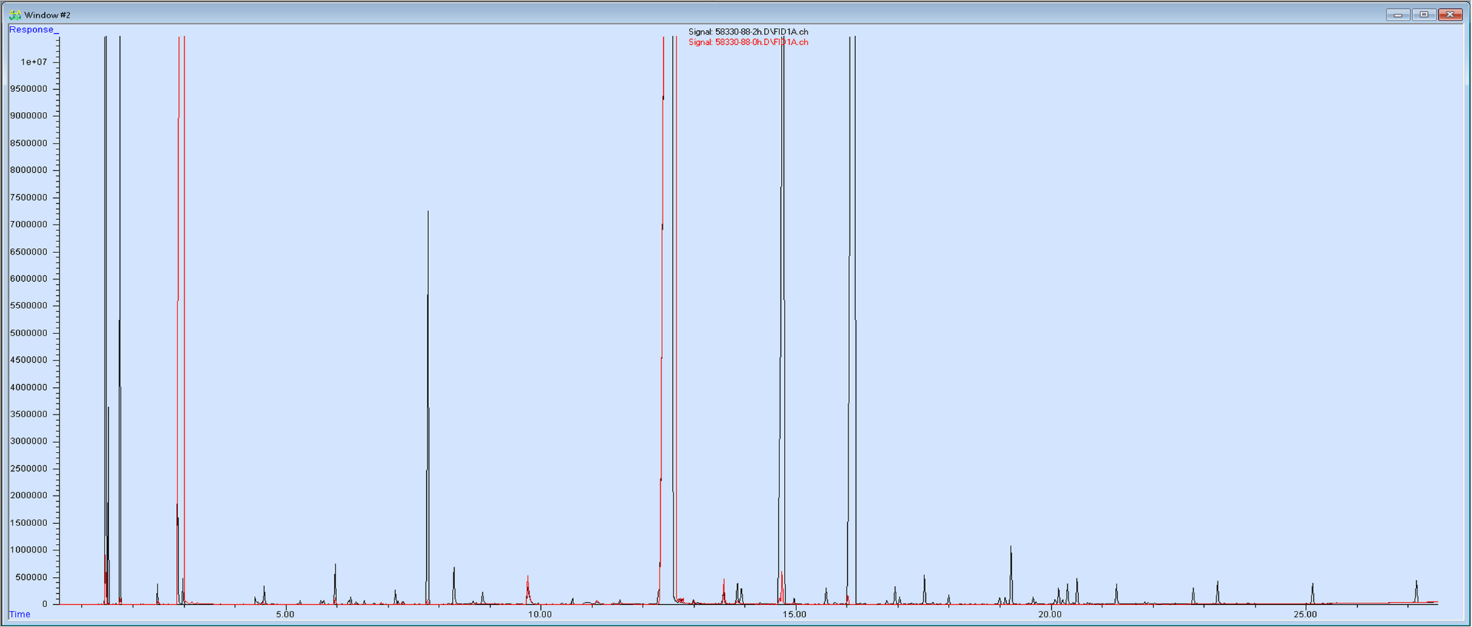


**Figure S11**: Reaction mixture from entry 3, Table 2 (neat Molyvan and dodecanol)


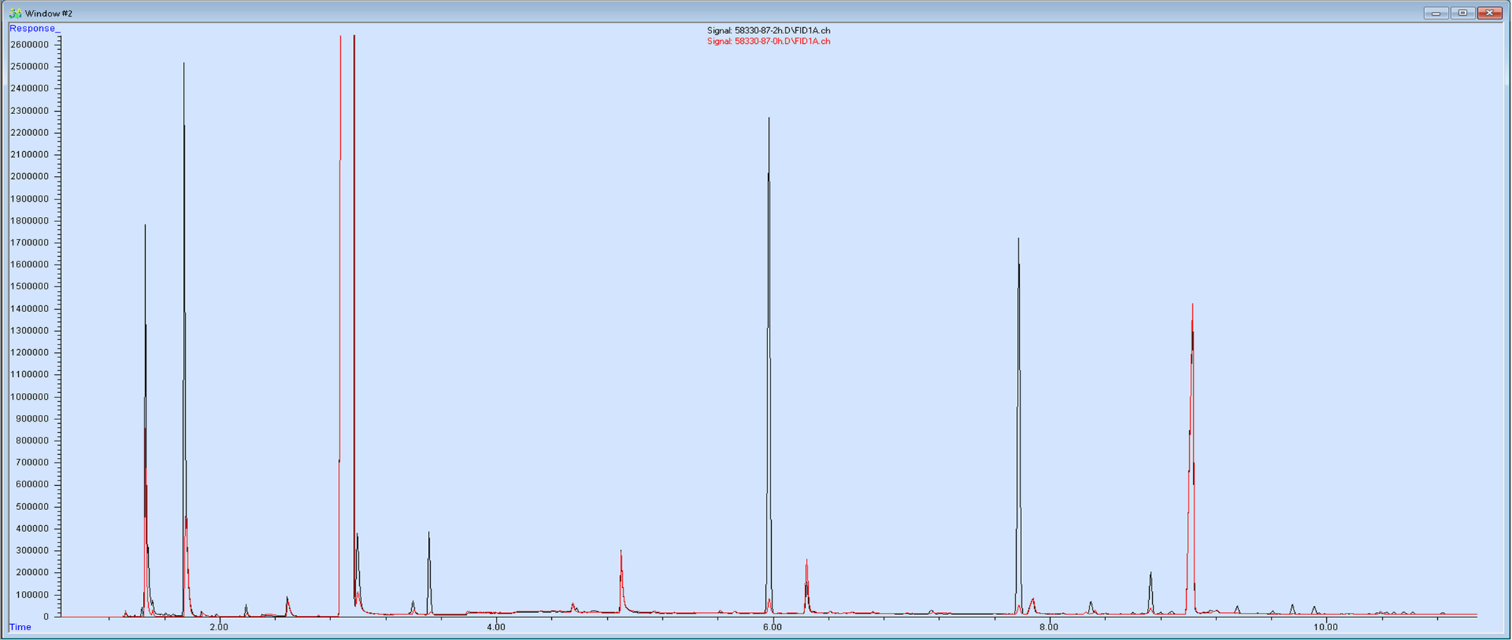


**Figure S12**: Reaction mixture from entry 4, Table 2 (neat Molyvan and methoxy PEG350)


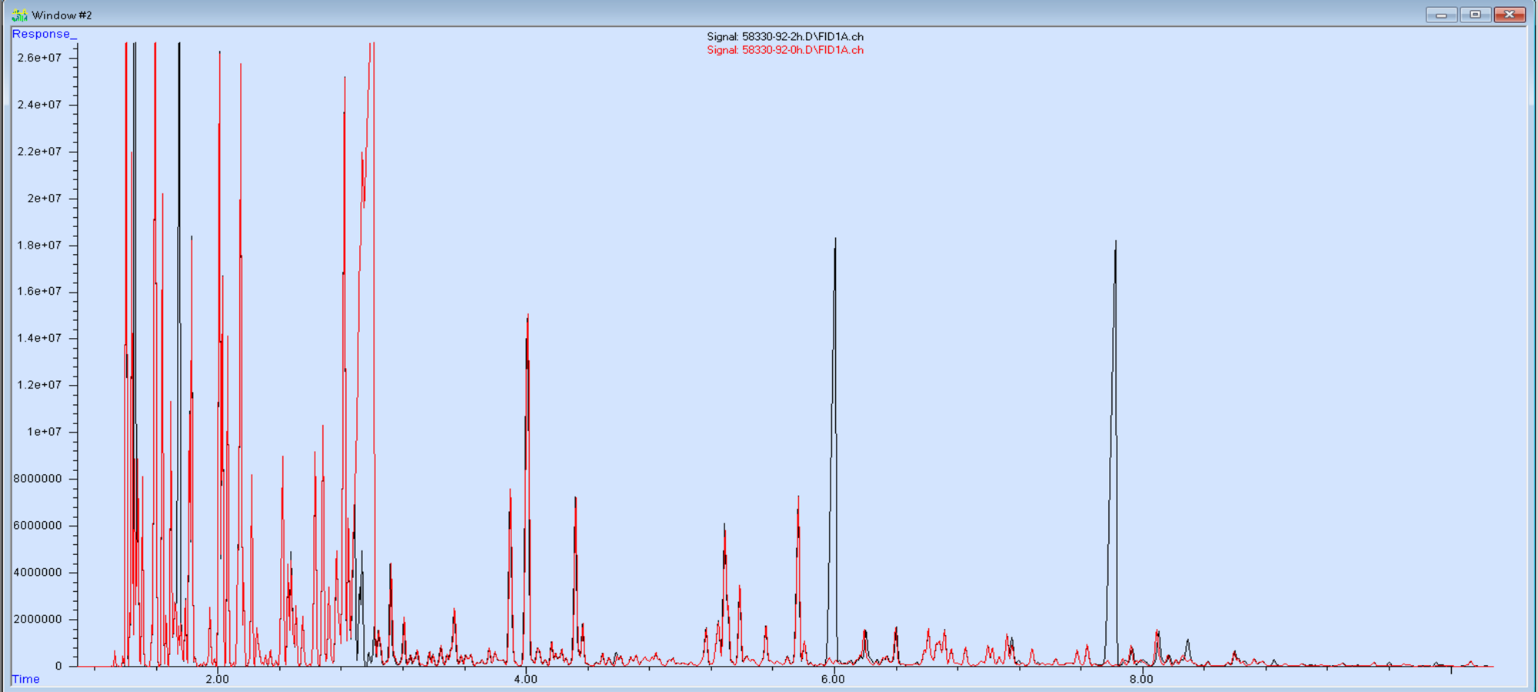


**Figure S13**: Reaction mixture from entry 5, Table 2 (neat Molyvan L and BOB3)


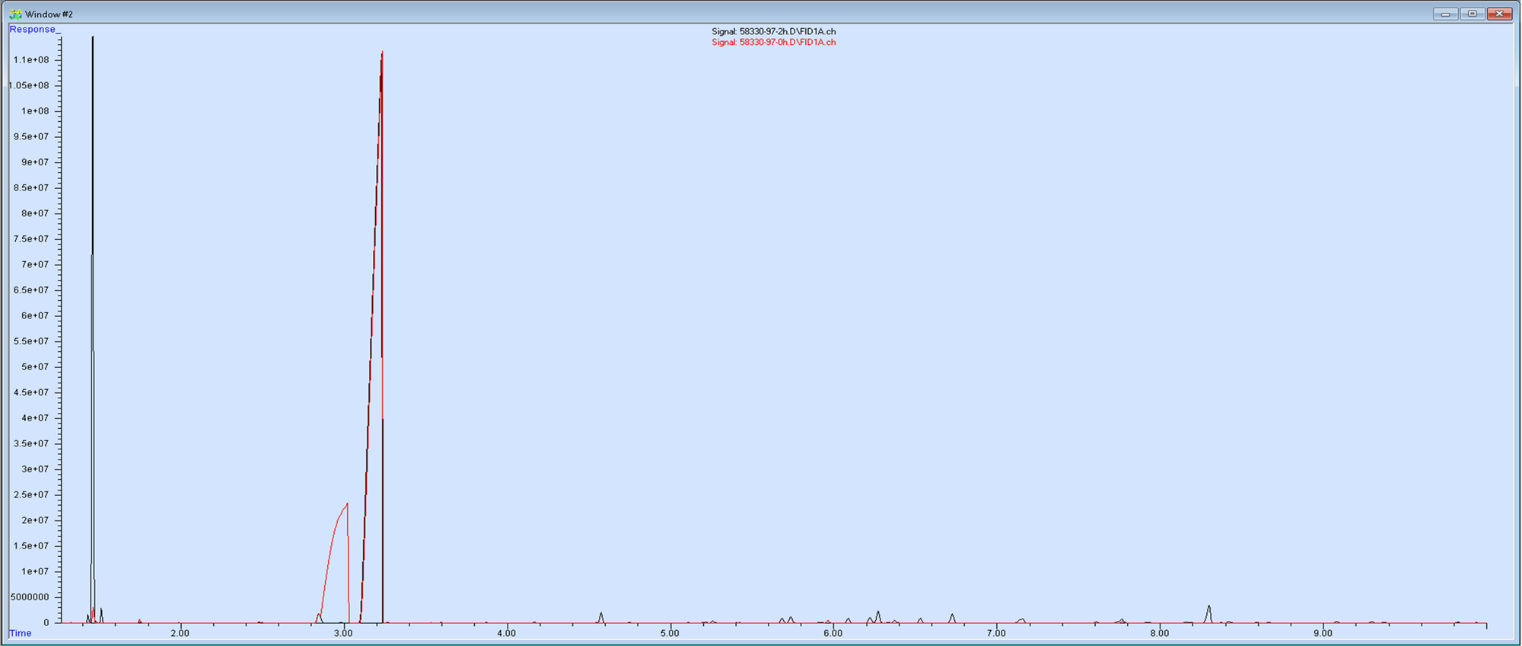


**Figure S14**: Reaction mixture from entry 6, Table 2 (neat Molyvan L and n-octane)


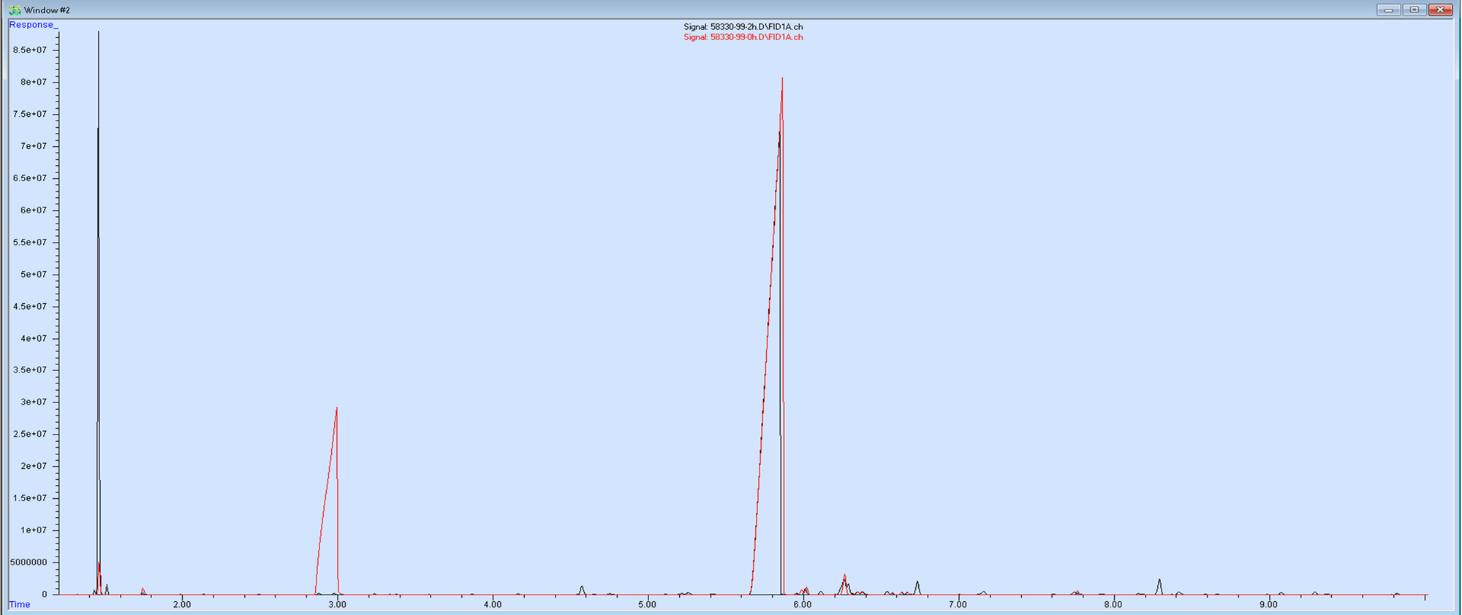


**Figure S15**: Reaction mixture from entry 7, Table 2 (neat Molyvan L and isooctane)

**Procedure for ^1^HNMR quantification experiments**

In addition to information provided in the manuscript, below are the exact amounts of samples tested and NMR plots with integration versus internal standard. A stock solution of CDCl_3_ was prepared and stored in the freezer between uses. A 100mg sample of DMTP was dissolved in CDCl_3_, to make a 13.01g solution. The so prepared stock solution was utilized to make NMR samples of the reaction mixture.

*Octane (1% Molyvan L in lubricant, entry 6, Table 1)*

Amount of rxn mixture: 0.2027g

Amount of CDCl_3_ standard solution = 0.6043g (equivalent to 4.64mg or 0.0237mmol of internal standard)

Considering the integration of the aromatic standard (4H), while the olefinic peaks at ~6.5ppm account for 1H (isoprene), the moles of isoprene in the sample is are 14.39 times higher than the standard, which equates to 0.34mmol of isoprene in 0.027g of rxn mixture. Theoretical amount of isoprene considering all prenol converts 100% to isoprene only is 3.35g in the whole mixture. Theoretical weight of the reaction mixture at beginning of reaction, ignoring Mo catalyst weight is 15.955. The total amount of isoprene in the rxn mixture is 26.7mmol, with a theoretical yield of 49.22mmol based on 5mL prenol starting material.

**Figure S16**. **^1^**HNMR and integration of Octane reaction (1% Molyvan L in lubricant, entry 6, Table 1)

*Octane (neat Molyvan L, entry 6, Table 1)*

A similar protocol as above was applied to the other octane mixture prepared with neat Molyvan L catalyst. The relevant amounts of NMR mixture evaluated and corresponding plot are shown below.

Amount of reaction mixture: 0.238g

Amount of internal standard and CDCl_3_ = 0.576g (equivalent to 4.42mg or 0.02278mmol of internal standard)

**Figure S17**. ^1^HNMR and integration of octane reaction (neat Molyvan L, entry 6, Table 2)

**Figure S18**: ^1^HNMR of isoprene in the reaction mixture before heating

**Figure S19**: ^1^HNMR of isoprene in the reaction mixture after heating

Table for author’s reference

| **Tale 1** |  |
| --- | --- |
| Entry #/NB# | Solvent |
| **1** 63B | Fuel Surrogate |
| **2** 75A | Dodecane |
| **3** 84 | Dodecanol |
| **4** 83 | Methoxy PEG350  Bottom layer |
| **4’** 83 | Methoxy PEG350  Top layer |
| **5** 91 | BOB3 |
| **6** 96 | N-octane |
| **7** 98 | isododecane |
| **8** 105 | PAO4 |
| **Table 2** |  |
| Entry#/NB# | solvent |
| **1** 89 | Fuel Surrogate |
| **2** 86 | Dodecane |
| **3** 88 | Dodecanol |
| **4** 87 | Methoxy PEG350 |
| **5** 92 | BOB3 |
| **6** 97 | n-Octane |
| **7** 99 | isododecane |
